# Using Stool Antigen to Screen for *Helicobacter pylori* in Immigrants and Refugees from High Prevalence Countries Is Relatively Cost Effective in Reducing the Burden of Gastric Cancer and Peptic Ulceration

**DOI:** 10.1371/journal.pone.0108610

**Published:** 2014-09-30

**Authors:** Thomas R. Schulz, Emma S. McBryde, Karin Leder, Beverley-Ann Biggs

**Affiliations:** 1 Victorian Infectious Diseases Service, Royal Melbourne Hospital, Parkville, Victoria, Australia; 2 Department of Medicine, University of Melbourne, at the Doherty Institute, Melbourne, Victoria, Australia; 3 Department of Epidemiology and Preventive Medicine, School of Public Health and Preventive Medicine, Monash University, Victoria, Australia; 4 Centre for Population Health, Burnet Institute Prahran, Victoria, Australia; Indian Institute of Science, India

## Abstract

**Objectives:**

Refugees and immigrants from developing countries settling in industrialised countries have a high prevalence of *Helicobacter pylori* (*H. pylori*). Screening these groups for *H. pylori* and use of eradication therapy to reduce the future burden of gastric cancer and peptic ulcer disease is not currently recommended in most countries. We investigated whether a screening and eradication approach would be cost effective in high prevalence populations.

**Methods:**

Nine different screening and follow-up strategies for asymptomatic immigrants from high *H. pylori* prevalence areas were compared with the current approach of no screening. Cost effectiveness comparisons assumed population prevalence's of *H. pylori* of 25%, 50% or 75%. The main outcome measure was the net cost for each cancer prevented for each strategy. Total costs of each strategy and net costs including savings from reductions in ulcers and gastric cancer were also calculated.

**Results:**

Stool antigen testing with repeat testing after treatment was the most cost effective approach relative to others, for each prevalence value. The net cost per cancer prevented with this strategy was US$111,800 (assuming 75% prevalence), $132,300 (50%) and $193,900 (25%). A test and treat strategy using stool antigen remained relatively cost effective, even when the prevalence was 25%.

**Conclusions:**

*H. pylori* screening and eradication can be an effective strategy for reducing rates of gastric cancer and peptic ulcers in high prevalence populations and our data suggest that use of stool antigen testing is the most cost effective approach.

## Introduction

Estimates suggest that half of the world's population is infected with *H. pylori*. Sero-prevalence studies in lower and middle income countries show rates of exposure above 80% [1]. *H. pylori* infection is usually acquired in childhood [1]. Colonisation persists for decades and is potentially lifelong, leading to chronic gastrointestinal inflammation, and stomach and duodenal ulcers.

*H. pylori* is also a major causative agent in the development of gastric cancer [1], and cancer occurs in 0.1–3% of those chronically infected [1]. Gastric cancer is the second most common cause of cancer death worldwide leading to 736,000 deaths annually [2], including 11000 in the United States [3]. The mean 5 year net cost of a patient with gastric cancer is over $50000 [4], and the five year survival rate is less than 20% [5]. Eradication of *H. pylori* has been shown to reduce progression to precancerous changes in the stomach [6], and to reduce the risk of developing gastric cancer by approximately one third [7].

Refugees and immigrants settling in western countries often have high rates of *H. pylori* infection (72–93%) [8], [9], as compared to the local population [8]. In Canada, overseas birth and immigration after 20 years of age were both shown to be risk factors for *H. pylori* infection [10]. Mexico, the largest source country for US immigration, has an *H. pylori* prevalence of 60% in serological surveys of 20 year olds [11]. More than 80% of African refugee children in Australia have positive stool antigen tests on arrival [9].

Approximately 12.5% (38.5 million) of the US population were born overseas, of whom 85% come from low or middle income countries [12]. Currently, neither *H. pylori* nor gastric cancer screening are recommended for this group if asymptomatic, with most guidelines recommending testing based on symptoms. However, it is recognised that detection based on symptoms can miss a significant burden of *H. pylori* infection [8] and gastric cancer [13].

A number of testing modalities exist for the detection of *H. pylori*. Serology is widely available and has a sensitivity of 92% (25% IQR 85–96%) and specificity of 83% (25% IQR 73–92%) depending on the test kit used [14]. Antibody levels decline slowly after eradication of *H. pylori* infection so a positive serology result may reflect past rather than current infection. Stool antigen testing is relatively inexpensive, monoclonal enzyme immunoassay (EIA) testing has a sensitivity of 94% (95% CI 93–95%) and specificity of 97% (95% CI 96–98%) [15]. Breath testing is a rapid, non-invasive test with a high sensitivity (95%) and specificity (98%) but is more expensive [16]. Gastroscopy with biopsy and culture remains the gold standard for *H. pylori* detection but is costly and more logistically challenging.

In this study we hypothesized that screening for and eradication of *H. pylori* in high prevalence populations would be cost-effective. Our objectives were to model the effect of various *H. pylori* screening strategies on the incidence of gastric cancer and ulcer disease in populations with different prevalence's of infection, and to compare the relative cost-effectiveness of each strategy (including savings accrued through prevention of gastric cancer and reduced burden of ulcer disease).

## Methods

**Figure 1:**
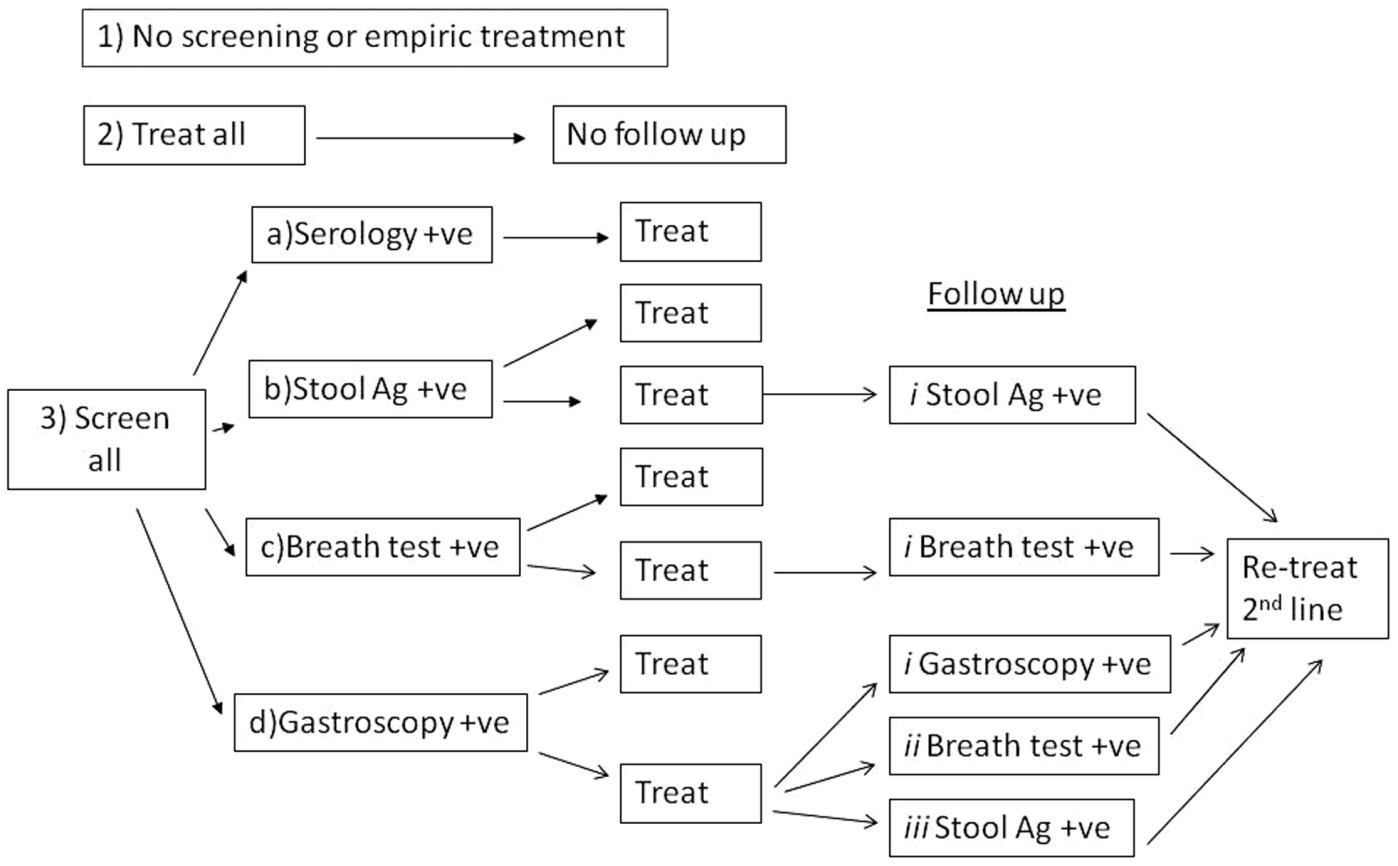
*H. pylori* management strategies included in the analysis.

Costs of test and treat strategies were compared to no screening and to empiric treatment strategies, both of which require no testing. Nine different screening and follow-up strategies were investigated (Figure 1).The empiric treatment approach was included as a comparator and may have a role in very high prevalence populations.

Costs were calculated as total costs for each strategy and benefit as the total cost of the outcome prevented. Primary benefits were the prevention of gastric cancer and the prevention of peptic ulcer disease, compared to no screening or treatment. Net costs were total costs minus savings accrued due to benefits of screening (ulcers and gastric cancers prevented).

The likelihood of a particular testing outcome was based on sensitivity and specificity values for the tests used (Table 1). Models were developed using decision analysis trees with the endpoints being total and net cost of the strategy and number of gastric cancers and ulcers prevented. Costs and treatment efficacy were based on published estimates (Table 1 & 2).

**Table 1:** Testing and treatment parameters used including estimated ranges around each parameter.

*Parameter*	*Best estimate*	*Lower range*	*Upper range*	*Distribution*	*Reference*
**Testing for ** ***H.pylori***					
**Breath test**					
Sensitivity (%)	95.3	92.2	97.5	95%CI^a^	[16]
Specificity (%)	97.7	94.8	99.3	95%CI	[16]
**Serology**					
Sensitivity (%)	92	85	96	IQR^b^	[14]
Specificity (%)	83	73	92	IQR	[14]
**Stool Antigen**					
Sensitivity (%)	94	93	95	95%CI	[15]
Specificity (%)	97	96	98	95%CI	[15]
**Gastroscopy with biopsy**					
Sensitivity (%)	95	90	99		[36]
Specificity (%)	99	95	100		[36]
**Treatment of ** ***H. pylori***					
HP7 efficacy	77%	27%	97%	full range	[29], [37]
2nd line treatment efficacy	90%	85%	95%		[38]
Sequential therapy efficacy	93%	91%	95%	95%CI	[37]
**Benefits of ** ***H.pylori*** ** eradication**					
Reduction in gastric cancers	RR^c^ 0.56	RR 0.4	RR 0.8	95%CI	[7]
Reduction in duodenal ulcers	RR 0.37	RR 0.26	RR 0.53	95% CI	[22]
**Other**					
Incidence of Peptic Ulcer disease	0.19%	0.10%	0.19%		[39]
Prevalence of Peptic Ulcer disease	1.50%	0.12%	1.50%		[39]
Incidence of duodenal ulcer if H. pylori +ve	5%	0.18%	17%	95% CI	[21]
**Treatment adverse effects (all)**					
Comparison	8%				[22]
Treatment	22%				[22]
**Cancer Survival (%)**					
1 year	41	39	42	95%CI	[5]
2 year	26	25	28	95%CI	[5]
3 year	21	19	22	95%CI	[5]
4 year	18	16	19	95%CI	[5]
5 year	16	14	17	95%CI	[5]

a CI  =  95% Confidence Interval.

b IQR  =  Interquartile range.

c RR  =  Relative Risk.

**Table 2:** Costs of testing and treatment for *H.pylori,* and costs of adverse outcomes associated with *H.pylori* in US dollars.

*Costs (in US$)*	*Original figure (US$)*	*Converted to 2011 (US$)*	*Reference*
**Testing and Treatment**			
*Physician visit*	86	86	[40]
*Serology*	29	30	[41]
*Breath test*	133	140	[41]
*Stool antigen*	21	22	[41]
*Eradication therapy*	355	373	[41]
*Gastroscopy with biopsy*	550	636	[21]
**Peptic ulcer annual cost**			
Peptic Ulcer Costs	866	1582	[42]
**Gastric Cancer costs**			
Mean cost of gastric cancer care (5 year net in 2004)			
Men	44203	52712	[4]
Women	41899	49965	[4]

Each model included the cost of the physician visits, which were assumed to be only for *H. pylori* management and not as part of other care or screening. It was assumed tests or empiric treatment (with a proton pump inhibitor (PPI), clarithromycin and amoxicillin), would be ordered during this initial visit. Strategies with a single time-point for testing were assumed to require up to two physician visits, with the second visit being needed only for those who required treatment because of positive testing. Strategies involving retesting were assumed to require up to three physician visits. Non-medical and indirect costs were not included, in keeping with other comparable modelling papers [5]. While a small proportion of individuals remain *H. pylori* positive after two courses of treatment, (the second course of treatment with PPI, bismuth, tetracycline and metronidazole) and require subsequent further testing and treatment, this scenario was not included. It is recognized that certain strains of *H. pylori* have higher risk of progression to gastric cancer [17]. As immigrants arrive from a multitude of countries from which strain prevalence varies or is not known, a standard risk of progression to cancer was used.

Costs were calculated in US dollars for 2011. Costs from earlier years were adjusted for inflation to bring them to 2011 values (Table 2). Analyses were performed for each strategy at three prevalence values (25%, 50% and 75%) with net costs per cancer prevented calculated for each strategy (Table 3). Total costs and numbers of cancers and ulcers prevented with each strategy (expressed per 1000 patients managed) were also calculated (Table 4).

**Table 3:** Net cost per cancer prevented (US dollars) for each strategy at varying prevalence rates of *H. pylori*.

Net cost per cancer prevented		Prevalence	
**Management Options**	*25%*	*50%*	*75%*
1) No screening	0	0	0
2) Treat all	477800	206900	116600
3) Screen and Treatment			
**a. Serology**			
No follow up	294700	169900	128300
**b. Stool Ag**			
No follow up	219200	142700	117100
i) Follow with stool Ag and retreat	193900	132300	111800
**c. Breath test**			
No follow up	360200	213800	165000
i) Follow with breath test and retreat	334600	216400	177000
**d. Gastroscopy**			
No follow up	972000	520600	370200
i) Follow up gastroscopy and retreat	939900	577200	456300
ii) Follow with breath test and retreat	820200	460100	340100
iii) Follow with stool Ag and retreat	794400	433900	313700

**Table 4:** Total cost (US dollars) and number of gastric cancers and ulcers prevented for each strategy for every 1000 people managed.

Prevalence	25%	25%	25%	50%	50%	50%	75%	75%	75%
**Management Options**	Total cost	Cancers prevented	Ulcers prevented	Total cost	Cancers prevented	Ulcers prevented	Total cost	Cancers prevented	Ulcers prevented
1) No screening	0	0	0	0	0	0	0	0	0
2) Treat all	458700	0.8	6.1	459000	1.7	12.1	459000	2.5	18.2
3) Screen and Treat									
**a. Serology**									
No follow up	280500	0.8	5.6	366600	1.6	11.2	452700	2.3	16.7
**b. Stool Ag**									
No follow up	226200	0.8	5.7	330700	1.6	11.4	435100	2.4	17.1
i) Follow with stool Ag and retreat	258000	1.0	7.6	393200	2.0	15.2	528300	3.0	22.8
c. Breath test									
**c. Breath test No follow up**	343000	0.8	5.8	449700	1.6	11.6	556400	2.4	17.3
i) Follow with breath test and retreat	404800	1.0	7.6	569800	2.0	15.3	734900	3.0	22.9
**d. Gastroscopy**									
No follow up	834300	0.8	5.8	942200	1.6	11.5	1050000	2.4	17.3
i) Follow up gastroscopy and retreat	1014800	1.0	7.6	1296700	2.0	15.3	1578600	3.0	22.9
ii) Follow with breath test and retreat	894400	1.0	7.6	1060900	2.0	15.3	1227400	3.0	22.9
iii) Follow with stool Ag and retreat	865900	1.0	7.6	1005000	2.0	15.2	1144100	3.0	22.8

Sensitivity analysis was performed on the most cost-effective strategy in the initial analysis (stool testing with retesting of those treated). The outcome measure of interest was the net cost per cancer saved. The parameters tested were cost of managing one cancer, cost of a physician visit, cost of medication for eradication, cost of managing one peptic ulcer and lifetime risk of gastric cancer. The change in net cost per cancer saved was estimated against the proportional change in each of the five parameters. A probabilistic model was developed in which the model parameters were drawn from their full uncertainty distributions, as given in Table 1. The distributions were assumed to be normal with the mean equal to the best estimate and upper and lower range equal to the 95% area under the curve of the normal distribution. For each of 10,000 iterations, a parameter was drawn from each uncertainty distribution and results calculated; including costs, number of cancers averted, number of ulcers averted false negatives and positive results. Sensitivity to change in parameters was estimated using multivariable regression, with cost per cancer saved as the continuous outcome variable and the parameters above as the predictor variables. Linear relationships were assumed and the parameters were not transformed. Figures represent the effect on cost per cancer prevented if each parameter was increased by 1% of the original estimate used.

## Results

**Figure 2:**
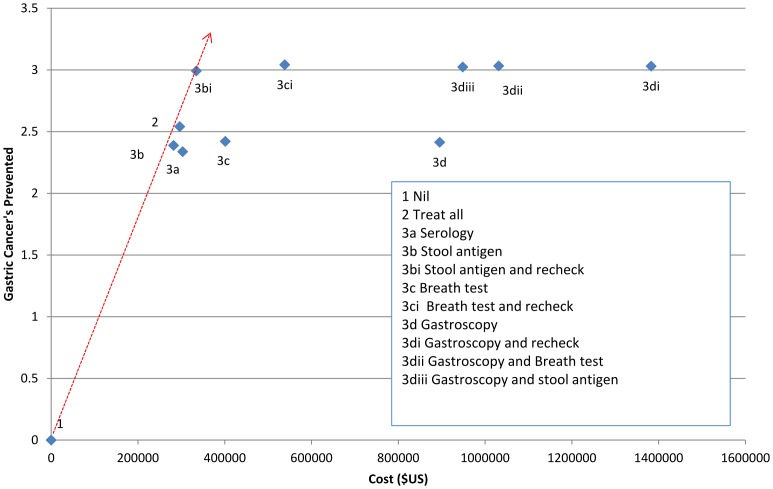
Net cost per number of cancers prevented for different strategies for screening and treatment of *H. pylori* in a population with a 75% prevalence of *H. pylori infection* shown as an Incremental Cost Effectiveness Ratio (ICER). Red line indicates lowest net cost per cancer prevented.

**Figure 3:**
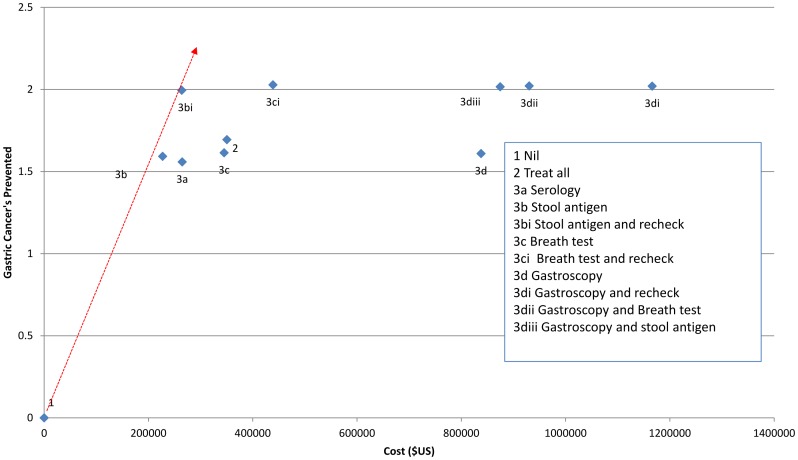
Net cost per number of cancers prevented for different strategies for management of *H. pylori* in a population with a 50% prevalence of *H. pylori infection* (ICER). Red line indicates lowest net cost per cancer prevented.

**Figure 4:**
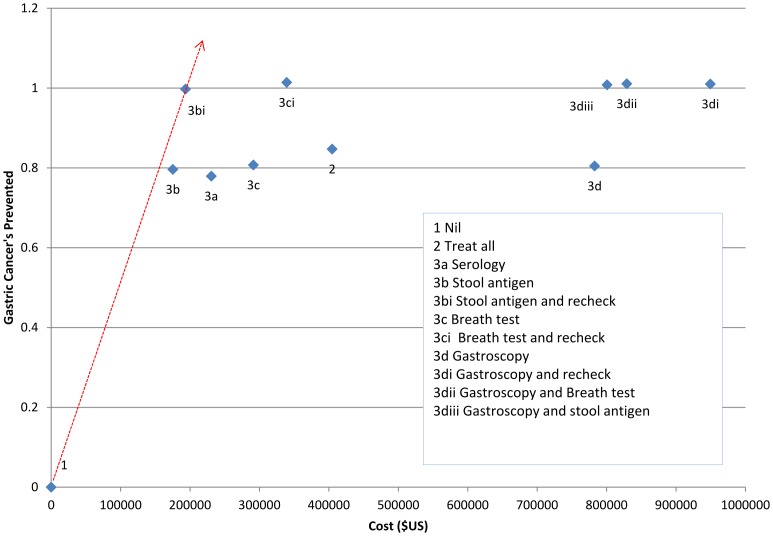
Net cost per number of cancers prevented for different strategies for screening and treatment of *H. pylori* in a population with a 25% prevalence of *H. pylori infection* (ICER). Red line indicates lowest net cost per cancer prevented.

For all three prevalence rates tested, the most cost effective approach relative to others was testing with stool antigen, with treatment for those who tested positive followed by retesting and further treatment if the initial treatment failed (Figure 2, 3, 4).

When the prevalence was assumed to be 75%, the estimated net cost per cancer prevented was $111800 (strategy 3b*i*) (Table 3). For every 1000 people managed under this strategy we expect that 3.0 gastric cancers and 22.8 ulcers would be prevented (Table 4). At 50% prevalence the net cost per cancer prevented was estimated to be $132300, with prevention of 2.0 cancers and 15.2 ulcers per 1000 people managed. At 25% prevalence the net cost per cancer prevented was estimated to be $193900, with prevention of 1.0 cancer and 7.6 ulcers per thousand people managed (Tables 3 & 4).

Treating all individuals without screening was also a relatively cost effective strategy in a high prevalence (75%) population, with a net cost per cancer prevented of $116600 (Table 3), although the overall number of cancers (2.5/1000 treated) and ulcers (18.2/1000 treated) prevented was lower than with a strategy involving retesting and further treatment (Table 4). At a lower prevalence estimate of 25% (Table 4) the cost of treatment became a very significant burden with the treat-all strategy because most of the population would receive unnecessary treatment. At 25% prevalence the strategy with the lowest cost of testing (stool antigen testing) offered the lowest overall cost, and post treatment testing and retreatment improved the net benefit.

The use of serology was more expensive at all prevalence levels tested and breath test, although having slightly better sensitivity and specificity than stool antigen, was considerably more expensive. Any strategy that involved the use of gastroscopy had considerably higher net costs per cancer prevented (Table 3) and total costs (Table 4). The net costs were slightly lower than the total costs indicating that the cost savings from preventing gastric cancer and ulcer disease contributed only a small component of the cost/benefit of each strategy.

**Figure 5:**
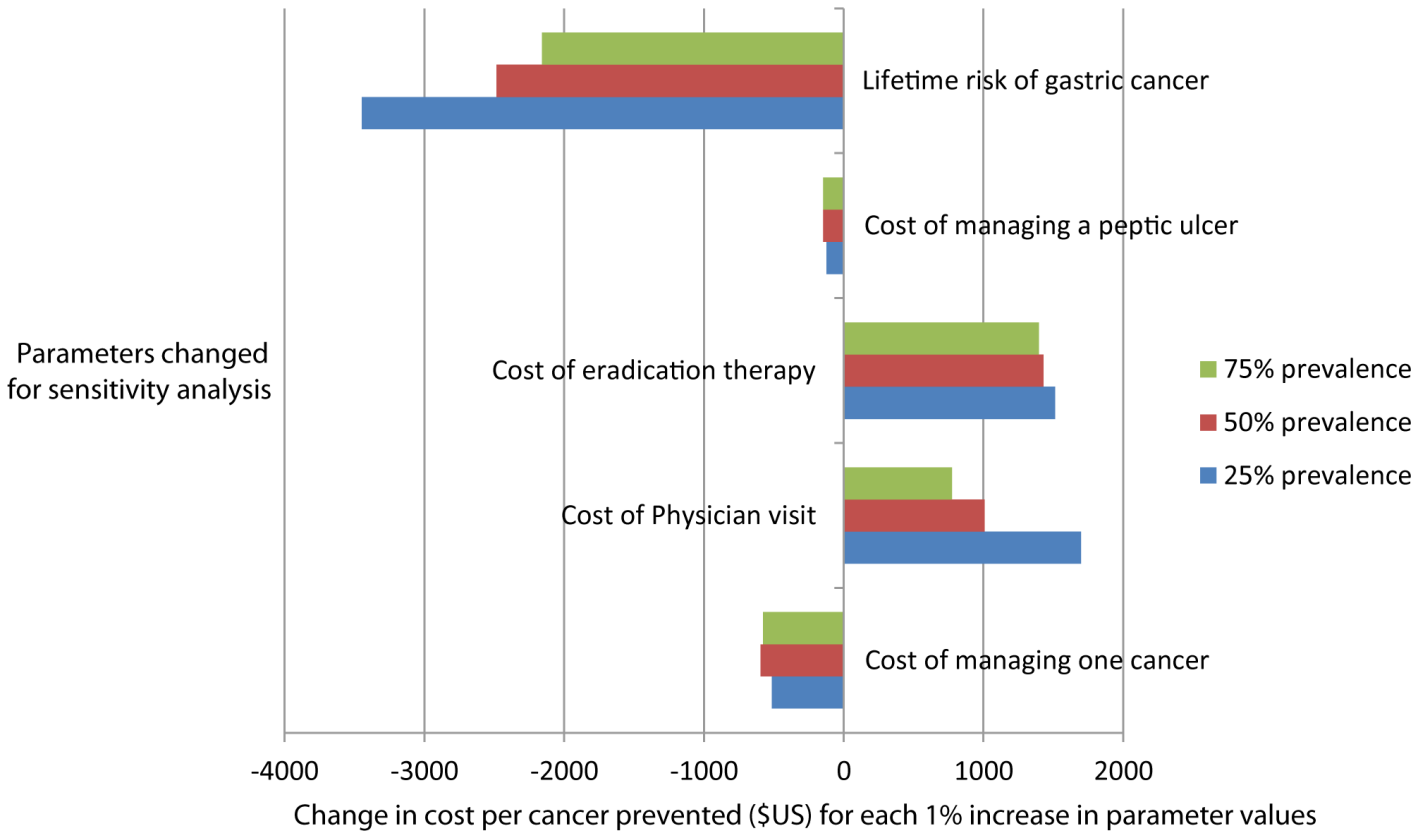
Sensitivity analysis for the most cost effective strategy (stool antigen with retesting). Horizontal bars represent the estimated net effect on cost per cancer prevented in US Dollars with a 1% increase in the listed parameters.

Figure 5 shows the sensitivity analysis for each of three prevalence values, using strategy 3bi, the optimal strategy. Parameters that represent the consequences of untreated *H.pylori* (peptic ulcer and gastric cancer) have negative values because as these costs rise, the value of eradicating *H.pylori* increases, the cost-effectiveness increases, and the net cost per cancer averted decreases. The parameters associated with the cost of the strategy have positive values, since as costs rise for any intervention, the cost per cancer averted rises. The cost of eradication therapy was the greatest cost associated with the strategy at high prevalence, while the risk of gastric cancer contributed significantly to the benefit of the strategy. Any increase in the estimated lifetime incidence of gastric cancer results in a significant decrease in the net cost per cancer prevented.

## Discussion

Screening and treatment of *H. pylori* in high-risk populations has been suggested as a means of reducing the burden of gastric cancer and peptic ulceration [8], [18] and been shown to be cost effective [19] however this is not routinely undertaken in Western countries [18]. Immigrants from developing countries represent a group with a high prevalence of *H. pylori* and hence a target group for screening strategies. Our modelling has shown that the costs associated with most of the available ‘test and treat’ strategies are not prohibitive. In particular, the use of a cheap and easily available stool antigen test has the potential to significantly lower the overall costs of screening, and deserves consideration in populations with high prevalence's of *H. pylori*. Notably the number of cancers and ulcers prevented is similar with stool antigen testing and retesting, breath test and retesting or any strategy involving gastroscopy and retesting. This indicates that the additional cost of more expensive screening strategies does not confer any significant additional benefit and reflects the similar sensitivity and specificity of these testing modalities.

Previous international modelling has shown that universal screening of 20 year olds for *H. pylori* is a cost effective way of reducing gastric cancer in a Chinese population [20]. Screening in lower prevalence populations has also been shown to be cost effective although the cost is significantly higher per quality adjusted life year (QALY) [5].

Our model may underestimate the benefits of screening and treatment as we did not include prevention of dyspepsia through *H. pylori* treatment and the associated reduced doctor visits for dyspepsia management, and potential for reduced hospital admissions [21]. The benefits of test and treat strategies may also be an underestimate as the cost of gastric cancer treatment may be considerably more than the cost estimates used in this analysis in patients over the age of 65 years [3]. The use of physician assistants or clinic nurses to order the initial testing would also lead to cost savings, and the use of more effective first line therapies could improve cost effectiveness. However, the frequency of treatment side effects, which reportedly occur in 22% of treated patients and 8% of placebo patients [22] needs to be considered and these additional side effects may increase costs.

*H. pylori* has been part of our gastrointestinal flora for 60000 years [23] so a recommendation for eradication should be made with caution. *H. pylori* prevalence rates have been falling in developed countries at the same time as allergic disease, reflux and obesity have been increasing [24]. Some randomised trial evidence demonstrates a small increase in weight after *H. pylori* eradication [25]. *H. pylori* eradication is also associated with a rise in prevalence of Barrett's Oesophagus [26], and increasing oesophageal cancer rates [27]. Concern that *H. pylori* eradication can lead to increased risk of gastro-oesophageal reflux disease (GERD) has not been confirmed in a large systematic review [28]. In any event this concern about oesophageal pathology is of small magnitude compared with the potential reduction in gastric cancer rates.

Antibiotic resistance to *H. pylori* is increasing and efficacy of standard treatment for *H. pylori* in many countries is now less than 80%, primarily due to clarithromycin resistance [29]. This is concerning for the implementation of a screening program. Other options that can be more effective include sequential therapy with PPI and amoxicillin for 5 days followed by PPI, clarithromycin and metronidazole for 5 days) [29], and longer courses (10–14 days) of quadruple therapy, including bismuth, tetracycline, metronidazole and a PPI [30].

Screening or empiric treatment for refugees and immigrants for infectious conditions is currently recommended for a number of pathogens. Empiric treatment for helminthic infections is cost effective and recommended in some settings [31]. Treatment costs for latent tuberculosis (TB) are over US$28000 (17,956 pounds) per episode of TB prevented [32]. TB in the United States now has a mortality less than 5% [33], compared to gastric cancer's 5 year mortality of 84% [5]. Screening and treatment for Hepatitis B virus is common in many immigrant groups, and is cost effective even at a population prevalence of less than 2% [34].

Stool sampling is currently routinely recommended for helminth detection for refugee groups arriving in many developed countries [35], and faecal antigen testing for *H. pylori* could be incorporated with stool testing for other pathogens, an additional important cost saving measure.

The current American College of Gastroenterology and also European guidelines do not recommend a general screen and treat strategy for *H. pylori* infection to reduce the risk of gastric cancer; and do not specifically address the issue of high risk populations [6], [36]. Asia Pacific guidelines, representing countries with a higher *H. pylori* prevalence, do recommend general screening for *H. pylori* in high risk populations although the strategy is not clearly defined [18].

Our data provide important evidence on which to base future recommendations.
